# The CARMA3-BCL10-MALT1 (CBM) complex contributes to DNA damage-induced NF-κB activation and cell survival

**DOI:** 10.1007/s13238-017-0441-3

**Published:** 2017-07-17

**Authors:** Shilei Zhang, Deng Pan, Xin-Ming Jia, Xin Lin, Xueqiang Zhao

**Affiliations:** 1 Department of Immunology, Tongji University School of Medicine, Shanghai, 200092 China; 2 Department of Basic Medical Sciences, Tsinghua University School of Medicine, Beijing, 100084 China; 3 Cancer Biology Program, Graduate School of Biomedical Sciences, The University of Texas, Houston, TX 77030 USA

**Dear Editor**,

Chemotherapy is one of major means for cancer treatments, and many of chemotherapeutic drugs are DNA damaging agents that reduce tumor growth through triggering cancer cell apoptosis or necrosis. Following DNA damage, ataxia telangiectasia mutated (ATM), a protein kinase, was activated and a cytosolic complex containing ATM, NEMO, RIP1 were formed (Biton and Ashkenazi, 2011). This ATM/NEMO/RIP1 complex cooperates to activate cytoplasmic adaptor protein TRAF6 (Hinz et al., 2010) and Ser/Thr kinase, TGF-β-activated kinase 1 (TAK1) and IKK complex to induce NF-κB activation through multiple signal transduction mechanisms depending on the severity of genotoxic stress and cell type (Wu et al., 2010). It has been shown that irradiation or chemotherapeutic drugs induce NF-κB activation through the functional IKK complex, but some reports suggest that UV irradiation activates NF-κB signal through IKK complex-independent but CK2-dependent manner (Kato et al., 2003). Of note, many DNA damaging agents can also activate NF-κB family of transcription factors that induce the expression of anti-apoptotic genes, thereby protecting cancer cells from apoptosis and resulting in the resistance of cancer cells to the chemotherapy. Although there are many studies on the mechanism of DNA damage-induced drug resistance, the molecular mechanism by which DNA damage induces NF-κB activation is not fully defined.

The caspase recruitment domain (CARD) and membrane-associated guanylate kinase-like domain protein (CARMA) family of proteins has three members, CARMA1, CARMA2, and CARMA3 that are encoded by three different genes (Blonska and Lin, 2011). CARMA proteins share the same set of structural domains, but a different pattern of tissue expression profile. Upon different stimuli, all CARMA proteins can form a complex with BCL10 and MALT1, and the CARMA-BCL10-MALT1 (CBM) complex functions to activate NF-κB signaling (Pomerantz et al., 2002; Grabiner et al., 2007; Jiang et al., 2011). Besides in NF-κB signaling, CBM complex is also involved in antiviral innate immune response by suppressing IRF3-type I IFN expression through inhibiting the formation of MAVS oligomerization in mitochondrion (Jiang et al., 2016). CARMA3 was reported to be relatively overexpressed in many tumor cells and associated with the malignant behavior of cancer cells (Li et al., 2012; Pan et al., 2016). It has been shown that CARMA3 and TRAF6 form a complex to mediating GPCR- and EGFR-induced NF-κB activation (Grabiner et al., 2007). Therefore, we hypothesized that the CBM complex contributes to DNA damage-induced NF-κB activation to protect genotoxic agent-induced cell death.

To determine the role of CBM complex in DNA damage-induced NF-κB activation, MEF cells isolated from Malt1 Het (+/−) or knockout (−/−) mice were stimulated with doxorubicin, a genotoxic reagent inhibiting the activity of topoisomerase II. Consistent with our hypothesis, NF-κB was potently activated by doxorubicin in control cells, but completely impaired in Malt1-deficient cells (Fig. 1A). To confirm that Malt1 is responsible to DNA damage-induced NF-κB activation, MEFs (Malt1^+/−^) and MALT-deficient MEFs (Malt1^−/−^) were treated with two other genotoxic reagent VP16 (etoposide) (10 μmol/L) and CPT (2 μmol/L), respectively. Consistently, we found that VP16- and CPT-induced NF-κB activation were defective in MALT1-deficient cells compared with controls (Fig. 1B), indicating that MALT1 is required for DNA damage-induced NF-κB activation. Since MALT1 forms a complex with CARMA1 and Bcl10 in hematopoietic cells, and CARMA3 and Bcl10 in non-hematopoietic cells to activate NF-κB, we examined the impact of CARMA3 or Bcl10 on DNA damage-induced signaling (Fig. 1C). CARMA3-deficient (Carma3^−/−^) and BCL10-deficient (Bcl10^−/−^) MEFs together with wild-type controls were treated with doxorubicin. Consistent with the result in Malt1 KO MEF cells, both CARMA3-deficient and BCL10-deficient cells were completely defective for NF-κB activation upon doxorubicin treatment. Similarly, we found that genotoxic reagents-induced NF-κB activation was significantly reduced in JPM50.6 cells (Fig. S1), in which the expression of CARMA1 is defective (Wang et al., 2002). Together, these results indicate that CBM complex mediates DNA damage-induced NF-κB activation in both hematopoietic and non-hematopoietic cells.

**Figure 1:**
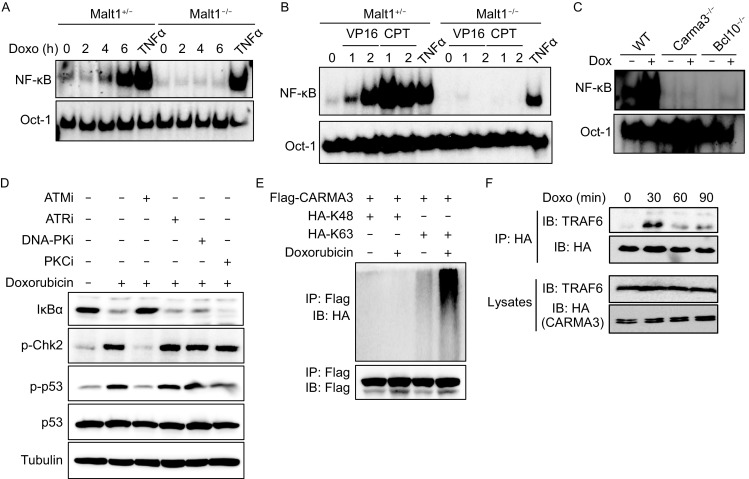
**The CBM complex is required for DNA damage-induced NF-**κ**B activation by chemotherapy agents**. (A) Early-passage (P1) MEFs isolated from Malt1 knockout (Malt1^−/−^) and control littermate (Malt1^+/−^) embryos were stimulated with doxorubicin (1.5 μg/mL) or TNF (10 ng/mL) for indicated periods. NF-κB activation and Oct-1 levels were determined by the gel shift assay. (B) Malt1^+/−^ and Malt1^−/−^ MEFs were stimulated with VP16 (10 μmol/L), CPT (2 μmol/L) or TNF (10 ng/mL) for indicated periods and the resulting cell nuclear lysates were determined by the gel shift assay. (C) MEFs from wild type, CARMA3^−/−^ (Card10^−/−^), and Bcl10^−/−^ embryos were isolated and stimulated with doxorubicin (1.5 μg/mL) for indicated periods. (D) Hela cells were either left untreated or pretreated with ATM, ATR, DNA-PK or PKC inhibitor for 1 h, following by stimulating with doxorubicin (1.5 μg/mL). Cellular lysates were analyzed by Western blot. NF-κB was detected using anti-IκBα antibody. DNA damage signaling was detected with p-p53 and p-Chk2 antibodies. Tubulin was used as loading controls. (E) Flag-CARMA3 was transfected with HA-ubiqutin K48 or HA-ubiqutin K63, respectively into 293T cells, following by left untreated or treated with doxorubicin (1.5 μg/mL). Cell lysates were immunoprecipitated with anti-Flag antibodies and then detected with indicated antibodies. (F) CARMA3-HA-reconstituted MEFs were treated with doxorubicin (1.5 μg/mL) for indicated time points and then immunoprecipitated with anti-HA antibodies. The resulting samples and lysates were analyzed by immunoblotting using the indicated antibodies

PKC family of kinases has been shown to play important roles in CBM complex-mediated NF-κB activation upon various stimulations through promoting phosphorylation of CARMA family proteins, which induces the association of CARMA proteins with the downstream signaling components, Bcl10 and Malt1 (Mahanivong et al., 2008). In addition, it has been reported that doxorubicin can activate PKC delta (PKCδ) (Diaz Bessone et al., 2011). Therefore, we hypothesize that the CBM complex is activated by PKC isoforms upon DNA damage. To test this hypothesis, we pretreated primary MEFs with PKC inhibitor GF109203X (PKCi) and induced NF-κB activation by either doxorubicin (1.5 μg/mL) or PMA (100 ng/mL). We found that PKC inhibitor efficiently blocked PMA-induced nuclear translocation of NF-κB (Fig. S2, lanes 7 and 10), but there was no obvious effect for doxorubicin-induced NF-κB activation (Fig. S2, lanes 6 and 9). To further confirm this finding, we treated HeLa cells with this PKC inhibitor (PKCi) following by doxorubicin treatment (Fig. 1D, lane 6). We found that the IκBα degradation was intact in the presence of the PKC inhibitor compared with controls (Fig. 1D, lanes 2 and 6), suggesting that DNA damage-induced NF-κB activation is independent on PKC family of kinases. These data indicate that PKC does not contribute to doxorubicin-induced NF-κB activation, and CBM complex might be activated through another undefined mechanism upon DNA damage.

Recent studies have suggested that ATM is required for NF-κB activation in response to various genotoxic reagents, but the role of DNA-PK and ATR, another two DNA damage sensors, in this activation remains controversial (Li et al., 2001). To determine the exact sensor in doxorubicin-induced NF-κB signaling, we tested the effect of ATM, ATR or DNA-PK inhibitors on NF-κB activation in response to stimulation of doxorubicin in HeLa cells (Fig. 1D). Consistent with previous study, doxorubicin-induced IκBα degradation was impaired by ATM inhibitor (Fig. 1D, lane 3), but not ATR or DNA-PK inhibitor (Fig. 1D, lanes 4 and 5). Consistent with NF-κB activation, phosphorylation of Chk2 and p53 was also defective upon ATM inhibitor treatment (Fig. 1D, lane 3) compared with the control. In contrast, the activity of chk2 and p53 were comparable in the ATR or DNA-PK inhibitor and DMSO treated HeLa cells (Fig. 1D, lanes 4 and 5). These results are consistent with previous findings that ATM is required for DNA damage-induced NF-κB activation.

Our previous studies show that overexpressed CARMA3 is K63-linked polyubiquitination, which may regulate NF-κB activation (Jiang et al., 2016). To determine how DNA damage regulates CBM complex, we co-expressed CARMA3 together with K48- and K63-linked ubiquitin, and found that DNA damage significantly induced K63-linked polyubiquitination (Fig. 1E). It has been shown that TRAF6 is involved in DNA damage-induced NF-κB activation (Hinz et al., 2010). Therefore, we hypothesized that CBM complex may also recruit TRAF6 in response to DNA damage-induced signaling. To efficiently isolate CARMA3-containing complex, we reconstituted CARMA3-deficient MEF cells with HA-tagged CARMA3, and then stimulated these cells with doxorubicin. We found that CARMA3 was inducibly associated with TRAF6 following doxorubicin treatment (Fig. 1F). These results suggest that the involvement of CBM in DNA damage-induced NF-κB is through the recruitment of TRAF6.

To determine whether CARMA3 also modulates DNA damage response-induced IFN signal, we detected the phosphorylation of TBK1 upon doxorubicin stimulation. Consistent with previous report, doxorubicin-induced TBK1 phosphorylation at the presence of ATM inhibitor, likely because the excessively released ssDNA in the cytoplasm can activate TBK1. However, no difference of TBK1 phosphorylation was detected in wild-type or CARMA3-deficient MEF cells (Fig. S3A). To further confirm this finding, we used CARMA3 knock-down HeLa cells silenced by CARMA3 shRNA to examined TBK1 activation. Consistently, signal-induced phosphorylation of TBK1 was comparable in both WT and CARMA3-knock-down HeLa cells (Fig. S3B). Together, these results indicate that CARMA3 is dispensable for DNA damage-induced TBK1 activation, although DNA damage-induced NF-κB activation is dependent on CARMA3.

Since CBM complex contributes to cell proliferation and survival by regulating growth factor-induced NF-κB pathway (Jiang et al., 2011; Pan et al., 2016), we hypothesized that CBM complex might also affect cell death induced by DNA damage response. To test this hypothesis, we treated WT and CARMA3 KO MEF cells with doxorubicin and examined the apoptosis of these cells, and found that there were more than 2 fold of dead cells in CARMA3-deficient MEFs compared with WT control cells following doxorubicin treatment for 6 h and 9 h, while no significant difference in the absence of doxorubicin treatment (Fig. 2A). Similarly, when we treated WT and Bcl10 KO MEF cells with doxorubicin, we found that significant more apoptotic cells in Bcl10 KO MEF cells than WT cells (Fig. S4). Together, these data suggest that CBM complex is necessary for pro-survival signaling to antagonize DNA damage-induced apoptosis.

**Figure 2:**
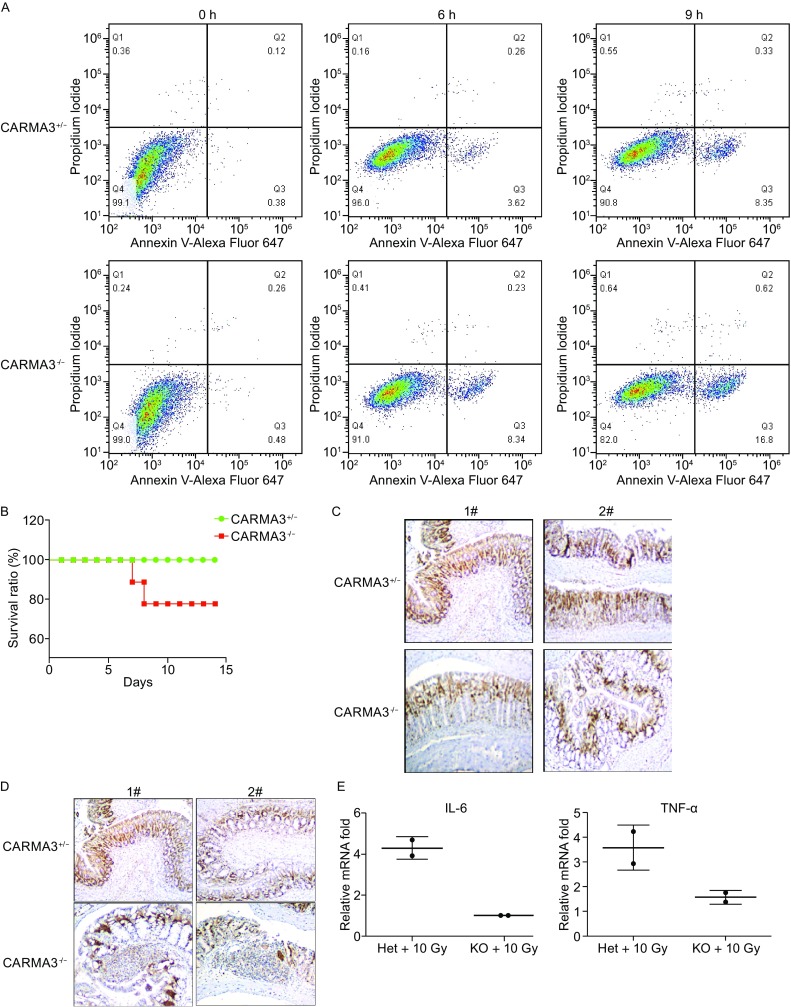
**CARMA3 affects doxorubicin-induced cell apoptosis** ***in vitro*** **and tissue repair upon irradiation induced DNA damage** ***in vivo***. (A) CARMA3^−/−^ MEFs were treated with doxorubicin (1.5 μg/mL) for 6 h and 9 h, respectively. Cells were collected and subjected to Annexin V/PI staining and analyzed by flow cytometry. (B) CARMA3^−/−^ mice and control littermate (CARMA3^+/−^) were exposed to 12 Gy abdominal irradiation. Mice survival was monitored for continuous 15 days. (C) 10 Gy abdominal irradiation treated CARMA3^−/−^ mice and control mice colons were collected at 3 days after irradiation. Representative staining for Ki67 indicating the cell proliferation were shown. (D) The infiltration of inflammatory cells in the indicated mice colons. (E) Real-time quantification of the expression levels of the indicated genes by qRT-PCR analysis from isolated colons, normalized against GAPDH expression

To examine the contribution of CARMA3 to DNA damage response *in vivo*, we monitored the survival of WT and CARMA3-deficient mice following irradiation at 12Gy. All mice in WT control group survived within 14 days after irradiation, while about 23% of CARMA3-deficient mice died (Fig. 2B). This result suggests that CARMA3 partially protected mice from irradiation effects. Because Ki67 staining was used as a proliferative marker of quiescent cells after irradiation, colons of WT or CARMA3-deficient mice were isolated and examined for Ki67-positive cells by immunohistochemical staining. We found that tissues from CARMA3^+/−^ mice had significantly more Ki67-positive staining than those from CARMA3^−/−^ mice (Fig. 2C), indicating the impaired tissue repairing in the absence of CARMA3. Moreover, more infiltration of inflammatory cells was found in CARMA3^−/−^ mice compared with control mice (Fig. 2D). Consistent with NF-κB activation *in vitro*, we found the reduced expression of NF-κB-dependent cytokines, IL-6 and TNFα, in colonic samples from CARMA3^−/−^ mice (Fig. 2E). Together, these data indicate that CARMA3 promotes tissue repair caused by irradiation-induced DNA damage response.

In summary, our data provides the genetic and biochemical evidence to reveal the molecular mechanism by which DNA damage activates NF-κB through CBM complex, therefore, providing a molecular basis for targeting the CBM complex to block DNA damage-induced NF-κB pathway. In addition, our results also highlighted the contribution of CARMA3 to the tissue repair and cell proliferation after irradiation.

## FOOTNOTES

This work was partially supported by grants from National Natural Science Foundation of China (Grant Nos. 31670904 and 81502460 to X.Z., and 81570211 to X.L). Shilei Zhang, Deng Pan, Xin-Ming Jia, Xin Lin, and Xueqiang Zhao declare that they have no conflict of interest. All institutional and national guidelines for the care and use of laboratory animals were followed.

## Electronic supplementary material

Below is the link to the electronic supplementary material.

Supplementary material 1 (PDF 720 kb)

## References

[CR1] Biton S, Ashkenazi A (2011). NEMO and RIP1 control cell fate in response to extensive DNA damage via TNF-alpha feedforward signaling. Cell.

[CR2] Blonska M, Lin X (2011). NF-kappaB signaling pathways regulated by CARMA family of scaffold proteins. Cell Res.

[CR3] Diaz Bessone MI, Berardi DE, Campodonico PB, Todaro LB, Lothstein L, de Kier Bal, Joffe ED, Urtreger AJ (2011). Involvement of PKC delta (PKCdelta) in the resistance against different doxorubicin analogs. Breast Cancer Res Treat.

[CR4] Grabiner BC, Blonska M, Lin PC, You Y, Wang D, Sun J, Darnay BG, Dong C, Lin X (2007). CARMA3 deficiency abrogates G protein-coupled receptor-induced NF-{kappa}B activation. Genes Dev.

[CR5] Hinz M, Stilmann M, Arslan SC, Khanna KK, Dittmar G, Scheidereit C (2010). A cytoplasmic ATM-TRAF6-cIAP1 module links nuclear DNA damage signaling to ubiquitin-mediated NF-kappaB activation. Mol Cell.

[CR6] Jiang T, Grabiner B, Zhu Y, Jiang C, Li H, You Y, Lang J, Hung MC, Lin X (2011). CARMA3 is crucial for EGFR-Induced activation of NF-kappaB and tumor progression. Cancer Res.

[CR7] Jiang C, Zhou Z, Quan Y, Zhang S, Wang T, Zhao X, Morrison C, Heise MT, He W, Miller MS (2016). CARMA3 is a host factor regulating the balance of inflammatory and antiviral responses against viral infection. Cell Rep.

[CR8] Kato T, Delhase M, Hoffmann A, Karin M (2003). CK2 is a C-Terminal IkappaB Kinase Responsible for NF-kappaB Activation during the UV Response. Mol Cell.

[CR9] Li N, Banin S, Ouyang H, Li GC, Courtois G, Shiloh Y, Karin M, Rotman G (2001). ATM is required for IkappaB kinase (IKKk) activation in response to DNA double strand breaks. J Biol Chem.

[CR10] Li Z, Qu L, Dong Q, Huang B, Li H, Tang Z, Xu Y, Luo W, Liu L, Qiu X (2012). Overexpression of CARMA3 in non-small-cell lung cancer is linked for tumor progression. PLoS ONE.

[CR11] Mahanivong C, Chen HM, Yee SW, Pan ZK, Dong Z, Huang S (2008). Protein kinase C alpha-CARMA3 signaling axis links Ras to NF-kappa B for lysophosphatidic acid-induced urokinase plasminogen activator expression in ovarian cancer cells. Oncogene.

[CR12] Pan D, Zhu Y, Zhou Z, Wang T, You H, Jiang C, Lin X (2016). The CBM complex underwrites NF-kappaB activation to promote HER2-associated tumor malignancy. Mol Cancer Res.

[CR13] Pomerantz JL, Denny EM, Baltimore D (2002). CARD11 mediates factor-specific activation of NF-kappaB by the T cell receptor complex. EMBO J.

[CR14] Wang D, You Y, Case SM, McAllister-Lucas LM, Wang L, DiStefano PS, Nunez G, Bertin J, Lin X (2002). A requirement for CARMA1 in TCR-induced NF-kappa B activation. Nat Immunol.

[CR15] Wu ZH, Wong ET, Shi Y, Niu J, Chen Z, Miyamoto S, Tergaonkar V (2010). ATM- and NEMO-dependent ELKS ubiquitination coordinates TAK1-mediated IKK activation in response to genotoxic stress. Mol Cell.

