# Supplementary material for: The CARMA3-BCL10-MALT1 (CBM) complex contributes to DNA damage-induced NF-κB activation and cell survival
Source: Protein Cell. 2017 Jul 17;8(11):856–60. doi: 10.1007/s13238-017-0441-3 (PMC5676590; doi:10.1007/s13238-017-0441-3)
Supplement: Supplementary file 1 — Supplementary material 1 (PDF 720 kb) [file 13238_2017_441_MOESM1_ESM.pdf]

The CARMA3-BCL1-MALT1 (CBM) complex contributes to DNA damage-induced NF-κB activation and cell survival

Shilei Zhang[1,2], Deng Pan[3], Xin-Ming Jia[1], Xin Lin[2,*], Xueqiang Zhao[2,*]

Supplementary Materials

Materials and methods

Cell Culture- HEK293T (human embryonic kidney cells) and HeLa (cervical cancer cells) cells were cultured in DMEM (HyClone) supplemented with 10% FBS. A549 (carcinomic human alveolar basal epithelial cell) cells were cultured with RPMI1640 medium containing 10% FBS. Primary wild type (WT) and CARMA3 knockout (KO), Bcl10 KO, Malt1 KO of mouse embryo fibroblast (MEF) cells were isolated from WT C57BL6 and respective KO mice and maintained in 5% CO2 at 37°C in DMEM medium supplemented with 10% FB (Gibico).

Antibodies and Reagents-Phosphorylation-specific antibodies to ATM and Chk2 were purchased from abgent (San Diego, USA). Antibodies against phosphorylated p53, phosphorylated TBK1, phosphorylated IRF3, phosphorylated ATM substrate, IκBα, TBK1 and IRF3 were purchase from Cell Signaling Technology (Danvers, MA, USA). Anti-p53, anti-tubulin and anti-Ki67 were purchased from Santa Cruz Biotechnology (Dallas, TX, USA). DNA-oligo probes for NF-κB (E3291) and Oct-1 (E3241) were purchased from Promega (Madison, WI, USA). Inhibitor against ATM, ATR, DNA-PK and PKC were purchase from Selleck.

Western Blot Analysis-The procedure from immunoblotting has been performed as described previously. Briefly, monolayer cells in 30mm dish were lysed in 100μl lysis buffer (250mM NaCl, 50mM HEPES at pH 7.4, 1mM EDTA, 1% NP-40, protease inhibitors) for 15 minutes. The lysates were centrifuged for 10min by 13000rpm at 4°C and then denatured at 95°C in 1XSDS loading buffer. 10 μl of the denatured protein sample was resolved on 10% SDS-PAGE gels, then transferred onto PVDF membranes (Bio-Rad) and probed with antibodies.

Transfection and Immuno-precipitation assay-For transfection, HEK-293T cells were seed in 30mm dishes at density of $2x10^6$. DNA plasmids were transfected by PEI as described previously. Thirty-six hours later, cells were lysed in 250 μl lysis buffer (150mM NaCl, 50mM HEPES at pH 7.4, 1mM EDTA, 1% NP-40, protease inhibitors) for 15 minutes. Then the cell lysates were collected by centrifuging at 13000rpm for 10min. 40μl of the lysates were denatured for western blot analysis as input control. 200μl of the lysates were incubated with 20μl protein A/G (abmart) and indicated

specific antibody for 3h at 4°C. After three times washes by RIPA lysis buffer, the precipitated proteins were suspended in 40μl lysis buffer containing 1xSDS loading buffer for western blot.

Quantitative real-time PCR (qPCR)-Total RNA was isolated from cells or tissues using TRIzol RNA isolation reagent (Invitrogen, Grand Island, NY,USA) and cDNA was generated using oligo-dT and SuperScriptII Reverse Transcriptase (Invitrogen) according to the manufacturer's protocol. Quantitative PCR was performed using SYBR Green PCR Master Mix with ROX(Genstar). The CT values for IL-6, IFN-β and TNF-α were normalized to GAPDH mRNA levels. The amplification primers used in the studies corresponded to: 5'-CCCTATGGAGATGACGGAGA-3' (forward), 5'- CTGTCTGCTGGTGGAGTTCA-3'(reverse) for mouse IFN-β ; 5'-ACTCACCTCTTCAGAACGAATTG-3'(forward), 5'-CCATCTTTGGAAGGTTCAGGTTG-3'(reverse) for human IL-6; 5'-ATGACCAACAAGTGTCTCCTCC-3'(forward), 5'-GGAATCCAAGCAAGTTGTAGCTC-3'(reverse) for human IFN-β; 5'-GGAGCGAGATCCCTCCAAAAT-3'(forward), 5'-GGCTGTTGTCATACTTCTCATGG-3'(reverse) for human GAPDH;

Animals and irradiation-All animal experimental protocols were approved by the Tsinghua University Animal Care Committee. Progeny homozygous for CARMA3$^{+/-}$ and CARMA3$^{-/-}$ mice with the same genetic background were bred separately for the experiments, and mice at the age of seven weeks were used. All animals were housed in modified barrier facility at the Tsinghua University Animal Center. For abdominal irradiation, mice were randomly divided into each group and were placed in a specially designed well-ventilated acrylic container and were exposed to 10 Gy of irradiation. After all mice were scarified at 3 days post irradiation, their colon samples were collected for immunochemistry staining with specific Ki67 antibody and for quantitative real-time PCR analysis of inflammatory cytokines. For survival ratio, CARMA3$^{+/-}$ and CARMA3$^{-/-}$ mice were exposed to 12 Gy of irradiation.

Statistical Analysis-At least two biological replicates were performed for all experiments unless otherwise indicated. Student's t test for paired observations was used for statistical analyses. Statistical significance was set at a p value of 0.05 or 0.01.

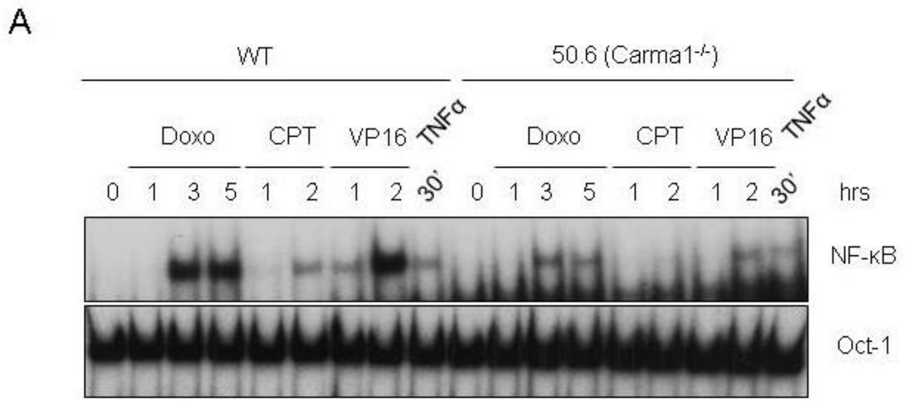

Fig S1. CARMA1 is required for doxorubicin-induced NF-κB activation. CARMA1 deficient Jurkat cells (50.6) and control cells were treated with doxorubicin (1.5μg/ml), VP16 (10μM), CPT (2μM), or TNF (10ng/ml) for indicated periods. NF-κB activation and Oct-1 levels were determined by the gel shift assay.

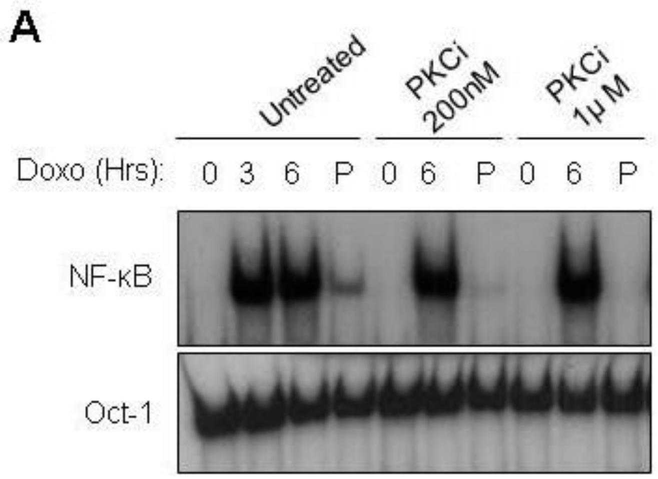

Fig S2. Doxorubicin induce PKC-independent NF-κB signaling activation. Primary MEFs were either left untreated or pretreated with PKC inhibitor (GF109203X) with indicated doses , following by stimulating with either doxorubicin (1.5μg/ml) or PMA (100ng/ml). NF-κB activation and Oct-1 levels were determined by the gel shift assay.

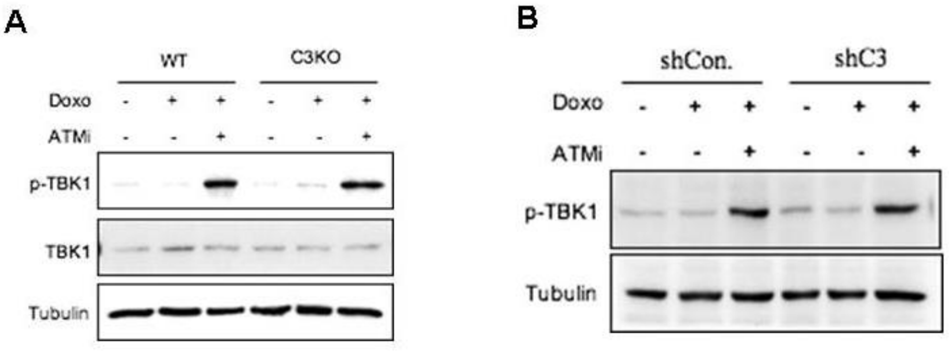

Fig S3. CARMA3 is dispensable for DNA damage-induced Interferon (IFN) signaling. (A) Primary MEFs from CARMA3$^{+/-}$ and CARMA3$^{-/-}$ mice were pretreated with ATM inhibitor KU-55933, and then stimulated with doxorubicin(1.5μg/ml) for 4 hours. Whole-cell lysates of MEFs were probed by western blot with the indicated antibodies. (B) CARMA3-knock-down Hela cells were treated with doxorubicin (1.5μg/ml) for 4 hours, with or without ATM inhibitor KU-55933 pretreatment. Cell lysates were prepared for western blot analysis of TBK1 activation.

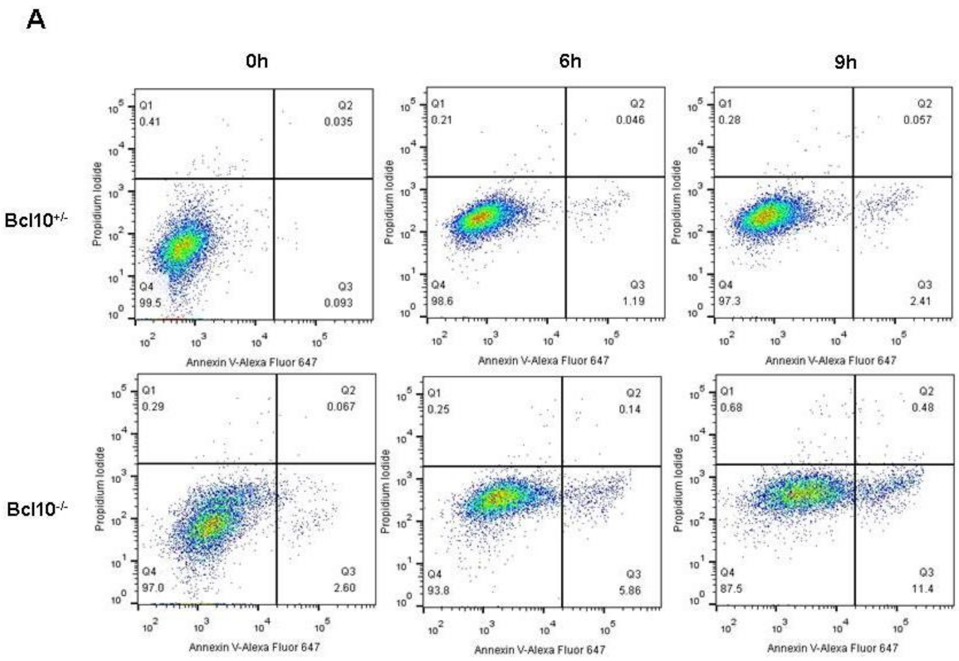

Fig S4. Bcl-10 deficiency sensitizes cells to doxorubicin induced apoptosis. Bcl-10$^{-/-}$ and control MEFs were treated with doxorubicin (1.5μg/ml) for 6 and 9 hours, respectively. Cells were collected and subjected to Annexin V/PI staining and analyzed by flow cytometry.
